# Co-Crystallization between Aliphatic Polyesters through Co-Inclusion Complexation with Small Molecule

**DOI:** 10.3390/molecules28104091

**Published:** 2023-05-15

**Authors:** Jia-Yao Chen, Xue-Wen Zhang, Tian-Yu Wu, Hai-Mu Ye

**Affiliations:** 1 Department of Materials Science and Engineering, College of New Energy and Materials, China University of Petroleum, Beijing 102249, China; 2 Beijing Institute of Space Launch Technology, Beijing 100076, China

**Keywords:** co-crystal, polyester, blend, inclusion complex, thermal property

## Abstract

Crystalline/crystalline blends of polymer have shown advantages in the preparation of new polymeric materials. However, the regulation of co-crystallization in a blend is still full of challenges due to the preferential self-crystallization driven by thermodynamics. Here, an inclusion complex approach is proposed to facilitate the co-crystallization between crystalline polymers, because the crystallization process displays a prominent kinetics advantage when polymer chains are released from the inclusion complex. Poly(butylene succinate) (PBS), poly(butylene adipate) (PBA) and urea are chosen to form co-inclusion complexes, where PBS and PBA chains play as isolated guest molecules and urea molecules construct the host channel framework. The coalesced PBS/PBA blends are obtained by fast removing the urea framework and systematically investigated by differential scanning calorimetry, X-ray diffraction, proton nuclear magnetic resonance and Fourier transformation infrared spectrometry. It is demonstrated that PBA chains are co-crystallized into PBS extended-chain crystals in the coalesced blends, while such a phenomenon has not been detected in simply co-solution-blended samples. Though PBA chains could not be totally accommodated in the PBS extended-chain crystals, their co-crystallized content increases with the initial feeding ratio of PBA. Consequently, the melting point of the PBS extended-chain crystal gradually declines from 134.3 °C to 124.2 °C with an increasing PBA content. The PBA chains playing as defects mainly induce lattice expansion along the *a*-axis. In addition, when the co-crystals are soaked in tetrahydrofuran, some of the PBA chains are extracted out, leading to damage to the correlative PBS extended-chain crystals. This study shows that co-inclusion complexation with small molecules could be an effective way to promote co-crystallization behavior in polymer blends.

## 1. Introduction

Blending is a popular way to regulate polymer properties and, thus, optimize materials with desired performance. Currently, a kind of common polymer blend is a crystalline/crystalline system, which consists of two components with different melting and crystallization temperatures, leading to diversified crystallization behaviors and crystal structures [1,2,3]. Meanwhile, the phase separation induced by the raising of mixing free energy and self-crystallization usually occurs, which significantly hinders the co-crystallization behavior in the blend. So, the manipulation of the property and performance of the crystalline/crystalline polymer blends is still facing great difficulties and the achievement of co-crystallization in the blends is still a challenge.

To realize effective co-crystallization in the polymer blends, some strategies have been proposed and verified. Blends of poly(3-hydroxybutyrate-*co*-3-hydroxyvalerate) (PHBV) and poly(3-hydroxybutyrate-*co*-3-hydroxypropionate) (PHBP) with the same crystallizable poly(3-hydroxybutyrate) (PHB) composition were confirmed to successfully gain co-crystallization [4], where PHBV chains could be incorporated into the thin PHBP crystals. Blends of homopolymer and its block copolymer with other compositions could be used to obtain the co-crystal between them [5,6,7], such as a blend of polyethylene (PE) and poly(vinyl cyclohexane)-*b*-polyethylene-*b*-poly(vinyl cyclohexane) (PVCH-PE-PVCH) [8] and a blend of poly(ethylene oxide) (PEO) and poly(ethylene oxide)-*b*-polybutadiene (PEO-PB) [9]. Additionally, pursuing the co-crystallization in a blend of isotopic polymers has also been carried out. An isotopic blend system consisting of a hydrogenated polymer and the deuterated counterpart could be employed to easily attain co-crystals in the whole composition [10,11,12,13], for example, a blend of hydrogenated PE (H-PE) and deuterated PE (D-PE) [14]. Even though the deuterated species shows a higher crystallization rate than the hydrogenated species [15], the similar chain structures provide thermodynamic stability and lead to the formation of co-crystals. Nevertheless, for the traditional all-hydrogenated polymer blends, the differentiation between components is much more significant. Consequently, co-crystallization is essentially difficult. Only limited cases of co-crystallization in homopolymer blends have been reported. The blends of polyamide 6 (PA6) and polyamide 4,10 (PA410) could form co-crystals only when the content of PA6 was lower than 20% [16]. In the blends of poly(butylene succinate) (PBS) and poly(butylene fumarate) (PBF), isomorphism could only be found in PBF-rich blends [17]. Forming of co-crystal in a polymer blend requires two aspects of good miscibility and kinetics advantage, which, however, usually could not be met during the crystallization from melt or solution.

The inclusion complex is a specified host/guest supramolecular structure in which polymer chains are isolatedly located as guest molecules in the channels of the host molecule [18,19]. When the host frameworks are removed, the as-released polymer chains will coalesce fast. Considering the indiscriminability between different polymer chains in the frameworks, co-inclusion complexation has been frequently used as a novel way to obtain polymer blends with particular characteristics [20]. Because of the good dispersion of polymer chains in the co-inclusion complex and the kinetics advantage during coalescence, many temporary miscible polymer blends were achieved even though they were immiscible in thermodynamics and could not be prepared via common process, including poly(ε-caprolactone) (PCL)/poly(l-lactic acid) (PLLA) [21], polycarbonate (PC)/poly(methyl methacrylate) (PMMA) [22], atactic poly(3-hydroxy butyrate) (*a*PHB)/PCL [23], poly(ethylene 2,6-naphthalate) (PEN)/poly(ethylene terephthalate) (PET) [24], PC/polystyrene (PS) [25], PC/PMMA/poly(vinyl acetate) (PVAc) [26] and PC/PET [27]. These as-coalesced blends displayed a single glass transition temperature (*T*_g_) during the first heating measurement and recovered to two distinct *T*_g_s in the follow-up measurement.

In our previous study, the co-inclusion complex of PLLA and poly(d-lactic acid) with thiourea was prepared, and it was found that the formation of stereocomplex crystals of PLLA/PDLA was significantly promoted when the guest chains were released and coalesced [28]. Thus, it is proposed that the formation of the co-crystal would be enhanced in a crystalline/crystalline polymer blend if the co-inclusion complexation approach is used. In this work, poly(butylene succinate) (PBS) and poly(butylene adipate) (PBA) were chosen as the model crystalline homopolymers to form co-inclusion complexes with urea. After washing away the urea, the PBS and PBA chains were released to coalesce, and the thermal properties and crystal structures of the coalesced PBS/PBA blends (PBS as the majority component) were systematically investigated. It is clearly demonstrated that the PBA chains were promoted to accommodate the PBS crystalline structure and impacted the properties of the crystal structure.

## 2. Results and Discussion

**Figure 1:**
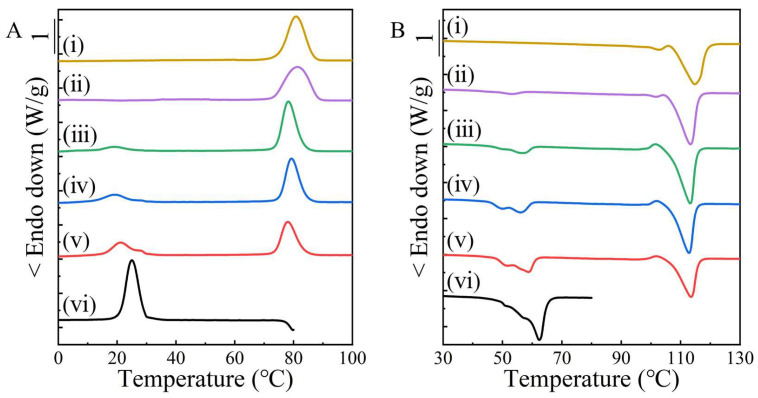
DSC scans of PBS, PBA and their simply-blended samples during the melt-cooling (**A**) and subsequent heating (**B**) processes at a constant rate of 10 °C/min, and (i)–(vi) indicate PBS, PBS/PBA-90/10, PBS/PBA-80/20, PBS/PBA-70/30, PBS/PBA-60/40 and PBA, respectively.

Conventional DSC investigation. A non-isothermal DSC measurement can reveal the crystal information and crystallization behavior of components in the polymer blends. As shown in Figure 1A for the simply-blended PBS/PBA group, neat PBS and neat PBA display melt-crystallization temperatures (*T*_c_s) at 80.9 °C and 25.0 °C, respectively. Thus, the exothermic *T*_c_s at around 80 °C in DSC curves ii–v were originated from the PBS components in the simply-blended samples; and those *T*_c_s, at around 19 °C in curves iii–v, were ascribed to the PBA components. The decline in *T*_c_ of PBA was due to the crystallization confinement caused by the PBS crystals [29]. The crystallization behavior of PBA in the 90/10 blend was too weak to be detected. The additional crystallization shoulder at the high-temperature side in the 70/30 and 60/40 blends could be stemmed from the fractionation crystallization of the PBA [2], indicating the reduction of confinement.

During the subsequent heating process (Figure 1B), the PBS components exhibited almost steady melting points (*T*_m_s) at about 114 °C, which was independent of the blend composition and close to that of neat PBS (114.8 °C). The PBA components in the blends displayed *T*_m_s within 50–60 °C, which were slightly lower than that of the neat PBA due to the confinement effect. So, both PBS and PBA should, respectively, form their own crystals in the blends. The crystal structure of PBS was hardly disturbed after being simply blended with the minority PBA, which was further confirmed in the X-ray investigation section.

**Figure 2:**
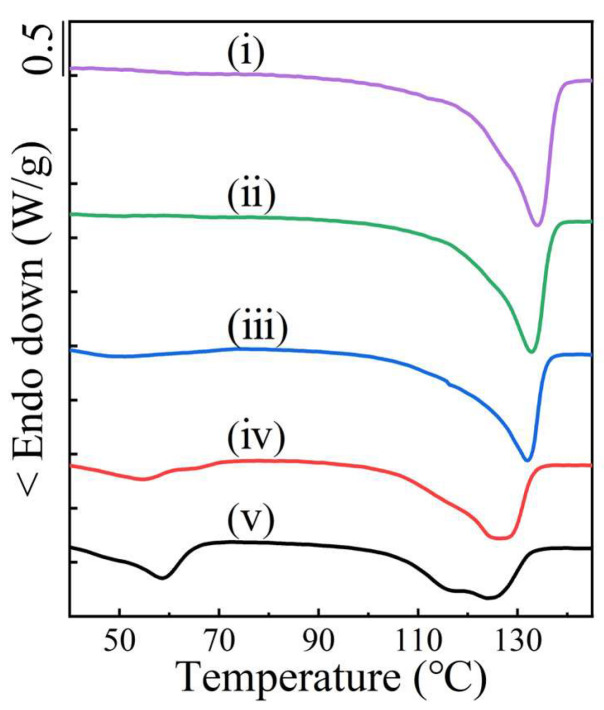
DSC heating curves of coalesced PBS/PBA samples at a rate of 10 °C/min, and (i)–(v) indicate PBS, PBS/PBA-90/10, PBS/PBA-80/20, PBS/PBA-70/30 and PBS/PBA-60/40, respectively.

The DSC curves of the as-prepared inclusion complex in Figure S1 just displayed an endothermic *T*_m_ at around 143 °C each, which were the same as the previously reported *T*_m_s of polyester/urea inclusion complexes [30]. There was not any melting trace of either PBS or PBA crystals. Thus, all polyester chains were well accommodated in the urea channels to form an inclusion complex, independent of the PBS/PBA ratio. After fast-washing the inclusion complexes with deionized water, the coalesced blends of PBS/PBA were obtained. Their thermal properties were investigated by DSC (Figure 2). The coalesced PBS crystals showed a *T*_m_ at 134.0 °C, which was significantly higher than the raw PBS and could be ascribed to the extended-chain crystals [30]. When some PBA was involved, the *T*_m_ of the PBS crystal dropped gradually in the coalesced PBS/PBA-90/10 (132.8 °C) and the coalesced PBS/PBA-80/20 (131.9 °C). With an increased content of PBA, the depression of *T*_m_ became more obvious. The coalesced crystals of PBS/PBA-70/30 and PBS/PBA-60/40 even displayed *T*_m_s at 126.1 °C and 124.0 °C, respectively, together with an apparent shoulder of 114 °C, which was assigned to the common lamellar crystals of PBS. That was, the increase of PBA not only lowered the *T*_m_ of the PBS extended-chain crystals but also led to the formation of lamellar crystals of PBS.

The DSC curves iii–v in Figure 2 all show a second lower *T*_m_ range of ~50–60 °C, which can be assigned to the melting of the PBA crystals. However, curve ii does not, indicating the absence of PBA crystals in the coalesced PBS/PBA-90/10 sample. Compared with the simply-blended samples (cf. Figure 1B), the lower melting enthalpy (Δ*H*_m_) demonstrated the remarkable depressing formation of the PBA crystal (Figure S2). There are two possible reasons for the decrease in PBA crystal content in the coalesced samples. (1) The PBA chains suffered stronger confinement from the surrounding PBS crystals, and (2) some PBA chains were co-crystallized into the PBS crystals. In the former case, the space between the PBS crystals was so small that the mobility of the PBA chains was restricted from forming crystals after coalescence. When the feeding content of the PBA was more than 10 mol%, the space between the crystals, after PBS coalescing, became larger, resulting in enough space for some PBA to crystallize.

Considering the crystallization kinetics advantage of released chains from the inclusion complex [31], the aggregation of the PBA chains for self-crystallization would be hindered. However, such a kinetics-advanced process could also benefit the retention of PBA chains in the PBS crystals, i.e., the formation of co-crystallization.

**Figure 3:**
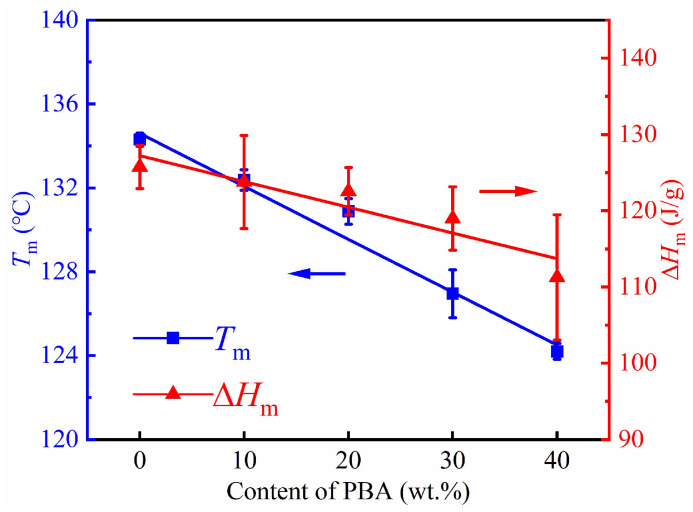
Melting temperature *T*_m_ and melting enthalpy Δ*H*_m_ (normalized by the mass fraction of PBS) for the indicated samples as a function of PBS content in coalesced PBS/PBA blends.

To confirm the above phenomena, the coalesced samples were repeatedly prepared and investigated four times. The data of *T*_m_ and the Δ*H*_m_ were processed and plotted in Figure 3. The *T*_m_ and normalized the Δ*H*_m_ of the PBS crystals respectively decreased from 134.3 °C and 125.7 J/g to 124.2 °C and 111.3 J/g in the coalesced sample of PBS/PBA-60/40, demonstrating that the increasing PBA chains were incorporated as defects in the PBS crystals. In addition, the formation of lamellar crystals of PBS, corresponding to the shoulder peak at ~114 °C, also contributed to the decrease in the Δ*H*_m_.

**Figure 4:**
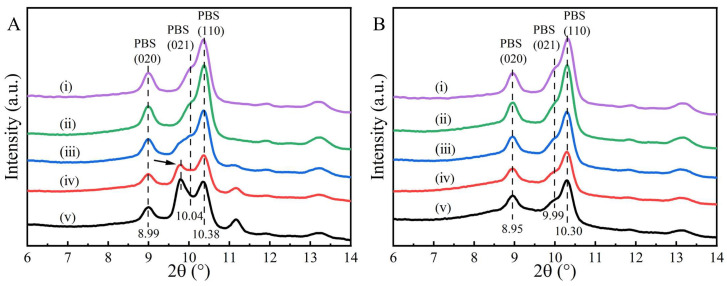
Wide angle X-ray diffractograms of simply-blended PBS/PBA samples at 26 °C (**A**) and 70 °C (**B**), and (i)–(v) indicate PBS, PBS/PBA-90/10, PBS/PBA-80/20, PBS/PBA-70/30 and PBS/PBA-60/40, respectively.

Crystalline structure analysis. X-ray diffractograms measured at room temperature (i.e., 26 °C) of the simply-blended samples are shown in Figure 4A. The distinct diffraction peaks at 2θ = 8.99°, 10.04° and 10.38° originate from the (020), (021) and (110) planes of PBS, respectively. The diffraction peak appearing at 2θ = 9.80°, indicated by a black arrow in the blends with a high PBA content, is ascribed to the (110) plane of PBA [2]. The positions of all diffraction peaks are unchanged when varying the PBS/PBA ratio, revealing the separate crystallization without co-crystallization.

To eliminate the overlapping of the PBA diffraction signal on PBS, the diffractograms of the simply-blended samples were measured at 70 °C (Figure 4B), at which temperature the crystals of the PBA component had been melted. The diffraction peaks of the PBS all shifted toward a low-2θ direction, resulting from the thermal expansion of the crystallographic planes at 70 °C in comparison to 26 °C [32]. However, the positions of the diffraction peaks of PBS still remain unchanged, with respect to the PBS/PBA ratio. Therefore, it was further confirmed that the PBA chains were not incorporated into the PBS crystals in the simply-blended samples.

**Figure 5:**
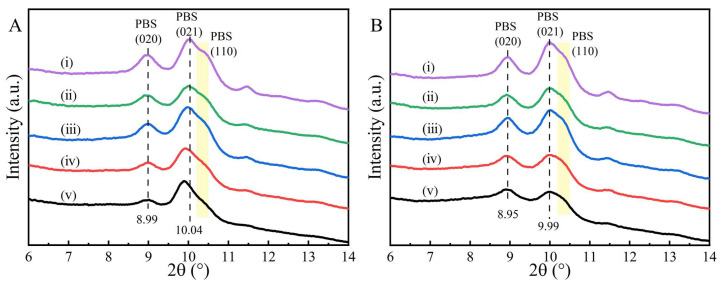
Wide angle X-ray diffractograms of coalesced PBS/PBA blend at 26 °C (**A**) and 70 °C (**B**), and (i)–(v) indicate PBS, PBS/PBA-90/10, PBS/PBA-80/20, PBS/PBA-70/30 and PBS/PBA-60/40, respectively.

Figure 5A shows the X-ray diffractograms of the coalesced blends measured at 26 °C. The apparent profiles seemed different from Figure 4A, which could be due to the broadening effect of the diffraction peaks for samples with smaller crystallite sizes. The shifting of peak position at 2θ = 10.04° in the profiles of the coalesced PBS/PBA-80/20, PBS/PBA-70/30 and PBS/PBA-60/40 should be originated from the overlapping effect of the PBA crystals.

**Figure 6:**
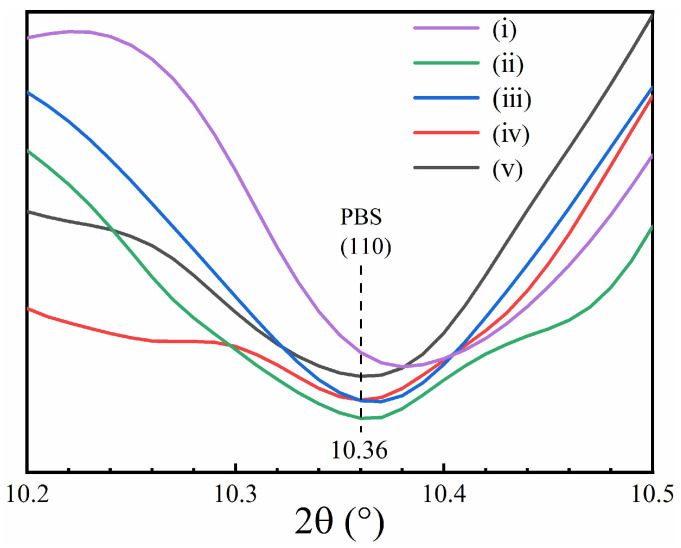
The 2nd derivatives of Wide angle X-ray diffractograms of coalesced PBS/PBA samples at 70 °C. (i)–(v) indicate PBS, PBS/PBA-90/10, PBS/PBA-80/20, PBS/PBA-70/30 and PBS/PBA-60/40, respectively.

To eliminate the overlapping effect from the PBA crystals, diffractograms of the coalesced blends were measured at 70 °C (Figure 5B). It was found that the peak positions of the (020) and (021) planes did not change after some PBA chains were incorporated into the PBS crystals (seen in the DSC investigation section). However, the diffraction peak shape of the (110) plane changed much obviously. To check whether the location of the (110) plane shifted or not, the 2nd derivatives of the diffractograms were figured out (Figure 6). The peak location of the (110) plane in the 2nd derivatives shifted from 2θ = 10.38° to 10.36°, revealing that the lattice expansion of the PBS crystals was along the [110] direction after it co-crystallized with the PBA. In combination with the unchanged positions of the (020) and (021) planes in all coalesced blends, it was reasonable to suggest that the co-crystallized PBA chains displayed as defects in the PBS crystals and contributed mainly to the lattice expansion along the *a*-axis.

**Figure 7:**
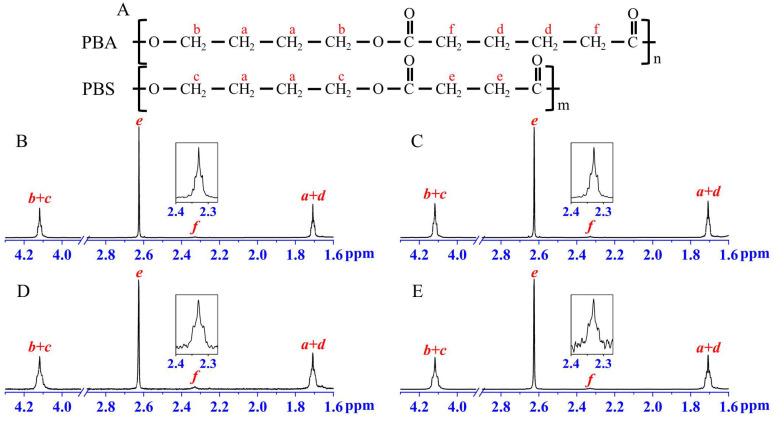
The chemical structures of PBA and PBS (**A**) and the ^1^H-NMR spectra of coalesced PBS/PBA-90/10 (**B**), PBS/PBA-80/20 (**C**), PBS/PBA-70/30 (**D**) and PBS/PBA-60/40 (**E**).

Soaking investigation. THF is a good solvent for PBA but a bad solvent for PBS. So, the coalesced blends were soaked in THF long enough (e.g., 24 h) to remove the PBA components as much as possible, the remaining structures were separated and dried in a vacuum oven at room temperature for 48 h for further measurement. The ^1^H NMR spectra of the remaining structures were employed to detect the chemical compositions. As shown in Figure 7, the characteristic nuclear magnetic resonance peak of the PBA at a chemical shift of 2.33 ppm still existed [33]. The mole ratios of the PBA/PBS in the THF-soaked blends were calculated based on the integrated areas of the two peaks at 2.33 ppm and 2.63 ppm, which increased with the initial feeding content of PBA, though much lower (Table 1). Consequently, the PBA chains were certainly confirmed to co-crystallize with PBS in the coalesced blends.

**Figure 8:**
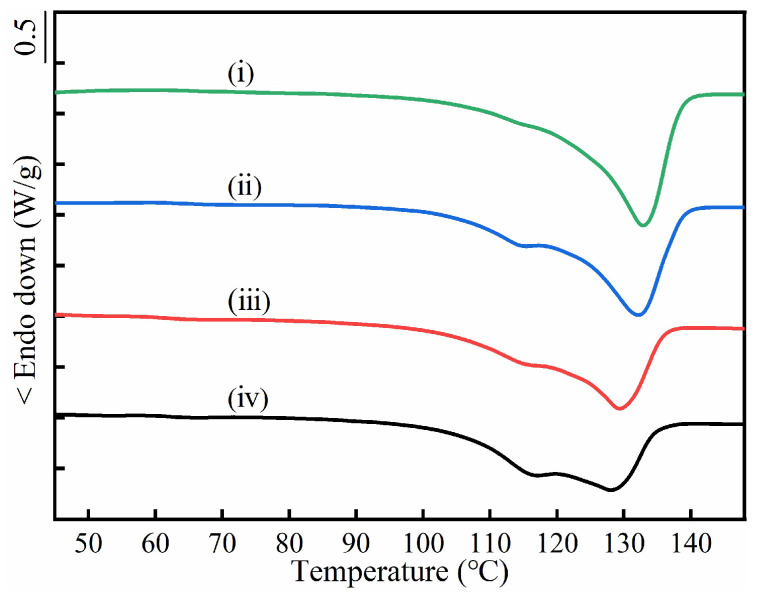
DSC heating curves of coalesced PBS/PBA samples at a rate of 10 °C/min after being soaked in THF. (i)–(iv) indicate PBS/PBA-90/10, PBS/PBA-80/20, PBS/PBA-70/30 and PBS/PBA-60/40, respectively.

The DSC investigation of the coalesced PBS/PBA blends after soaking was also carried out. As shown in Figure 8, no endothermic trace of PBA was detected and the melting signal of the PBS lamellar crystals at ~114 °C became much more apparent in comparison with the as-coalesced samples (cf. Figure 2). So, the lamellar crystals of PBS increased after the THF soaking treatment. To quantitatively evaluate the change of PBS lamellar crystal, the Lorentz peak decoupling process was used to fit the DSC curve to obtain the separate enthalpy (Δ*H*_m,l_), as shown in Figure S3. The values of the Δ*H*_m,l_ are tabulated in Table 2. It was clear that the Δ*H*_m,l_ increased after soaking, for example, the Δ*H*_m,l_ of the coalesced-PBS/PBA-60/40 was raised by ~32% (from 23.8 J/g to 31.5 J/g). The only plausible explanation for the increasing PBS lamellar crystal is that some PBS extended-chain crystals were destroyed and re-crystallized into lamellar crystals when part of the PBA chains, accommodated in the PBS extended-chain crystals, were extracted out during soaking in THF.

In addition, the *T*_m_ of the PBS extended-chain crystal increased by 0.1–4.4 °C after soaking (seen in Table 2), which meant that some of the extended-chain crystals with lower melting temperatures (e.g., containing a more defective PBA chain) had been damaged. The released PBS chains crystallized in the lamellar crystals and the released PBA chains were washed by the THF. The higher increasing degree of *T*_m_ of the PBS extended-chain crystals indicated the increased removal of the less stable extended-chain crystals, corresponding to the formation of extra PBS lamellar crystals. Considering the damage of PBS/PBA co-crystals during the soaking in THF, it is necessary to emphasize that the PBA content co-crystallized in as-coalesced PBS extended-chain crystals should be higher than the results shown in Table 1.

**Figure 9:**
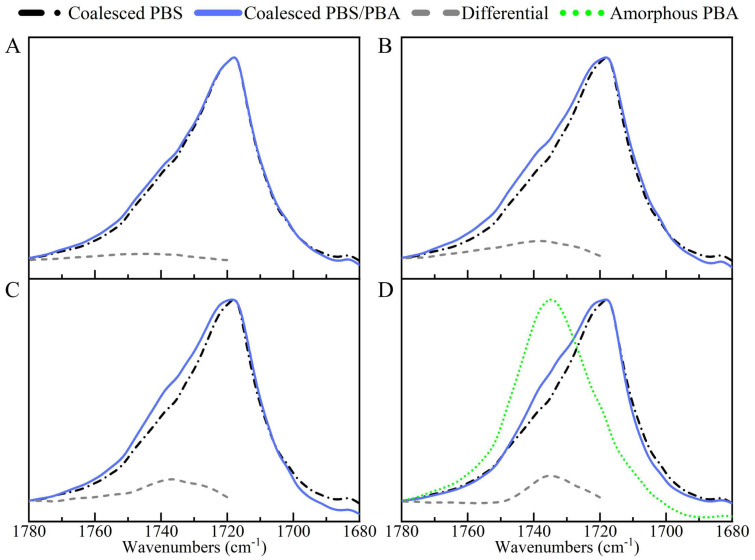
FTIR spectra of the coalesced PBS and PBS/PBA samples in the wavenumber range of 1780–1680 cm^–1^ after being etched by methylamine vapor for 48 h and corresponding differential spectra (grey dash line) between etched PBS (black dash dot lines) and PBS/PBA samples (blue solid lines). The blue solid lines in (**A**–**D**) indicate PBS/PBA-90/10, PBS/PBA-80/20, PBS/PBA-70/30 and PBS/PBA-60/40 after etching, respectively. The spectrum of amorphous PBA (green dot line) is inserted in (**D**) for comparison.

Methylamine Etching Experiment. Methylamine (MA) is active with polyesters and has the ability to clip the polyester chain structure because it can attack the carbonyl carbon atoms in the polyester chain. MA molecules convert the ester bond into an amide bond, thereby severing the molecular chain [34]. So, MA has been frequently used to remove the amorphous region for a better investigation of the crystalline structure and the properties of polyester [35,36]. Here, the coalesced PBS/PBA blends were etched with MA vapor at room temperature for an optimized time of 48 h. With such a long etching time, no separate PBA was left. The residual samples were washed, dried and then characterized by FTIR. As shown in Figure 9, the etched PBS showed a characteristic stretching vibration band of the crystalline carbonyl group (C=O) at 1719 cm^–1^. The absence of an amorphous C=O signal demonstrated that the sample had been etched sufficiently.

The spectrum of the coalesced PBS/PBA-90/10 after etching was almost the same as the etched PBS (Figure 9A) but showed slightly stronger absorption at the high-wavenumber side shoulder of the band. With the increase of the initial feeding ratio of the PBA, the signal of the shoulder became more apparent (Figure 9B–D). To obtain details, the differential spectra between the etched PBS/PBA and etched PBS were calculated. It was clear that a band at ~1736 cm^–1^ grew with the initial PBA content, which was close to that of C=O stretching vibrations of the amorphous PBA at 1735 cm^–1^. It was impossible for the existence of a free amorphous PBA after the MA etching. So, the remaining PBA could only locate the PBS crystalline structure, that is, the PBA chains were co-crystallized with the PBS. The slight shift of the PBA band might be due to the change in the surrounding chemical environment from the amorphous PBA (for the neat sample) to the crystalline PBS (for the coalesced sample).

## 3. Experimental

Materials. The Poly(butylene succinate) (PBS) was supplied by Xinjiang Blue Ridge Tunhe Sci.& Tech. Co. in Changji City, China and the Poly(butylene adipate) (PBA) was prepared through a two-step reaction of esterification and polycondensation in a melt state in the laboratory. Chloroform (AR grade), methanol (AR grade), tetrahydrofuran (THF, AR grade), hexafluoroisopropanol (HFIP, AR grade) and methylamine (40% aqueous solution) were all purchased from Shanghai Aladdin Reagent Co. in Shanghai City, China.

Both PBS and PBA were purified through a general procedure before being used. Polyester was first dissolved in chloroform to form a 5 wt% solution, then the solution was centrifuged at a rate of 10,000× *g* rpm for 20 min to remove any impurity. The clear solution was separated and precipitated with an excess amount of cold methanol, and the precipitates were collected and dried in a vacuum oven at 50 °C for 24 h. The viscosity-average molecular weights (*M*_η_) of PBS are determined as 89.0 kg/mol and the number-average molecular weights (*M*_n_) of PBA are determined as 38.0 kg/mol.

Preparation of polyester/urea inclusion complex. The solution for electrospinning was obtained by dissolving the PBS and PBA with different mole ratios and the urea together in HFIP with a total polyester concentration of 5% (*w*/*v*). The mole ratios of the urea to the repeated units of PBS and PBA were 0.83:0.17 and 0.85:0.15, respectively [30]. The electrospinneret was connected to a positive power of 15 kV and the receiver was connected to a negative voltage of −1.5 kV. The inner diameter of the spinneret was 0.6 mm and the distance between the spinneret and receiver was optimally chosen as 18 cm. The as-electrospun PBS/PBA/urea nanofiber mats were heated to 120 °C at a rate of 10 °C/min and held for 1 min to obtain their inclusion complexes.

Coalescence of polyester from inclusion complex. The inclusion complex samples were soaked in a large amount of deionized water and stirred at room temperature for 24 h to remove the urea. Then the coalesced PBS/PBA samples were dried in a vacuum oven at room temperature for 48 h before use.

Simply-blended PBS/PBA. The controlled samples for comparison were obtained through a solution-mixing process. The PBS and PBA were dissolved in HFIP at a concentration of 5% (*w*/*v*) with different proportions. The solutions were stirred at room temperature for 48 h, and then the solvent was evaporated to obtain the simply-blended PBS/PBA samples.

Differential scanning calorimeter (DSC) measurement. The thermal properties of the samples were measured on a NETZSCH 204F1 differential scanning calorimeter (DSC) equipped with an intercooler system under a nitrogen atmosphere. The instrument was calibrated with indium standard before measurement and operated with a constant flow (40 mL/min) of ultrapure nitrogen gas. All the samples were encapsulated in aluminum DSC pans, and each sample weighed about 5.0 mg.

Fourier transformation infrared spectrometer (FTIR) measurement. The samples were ground into a fine powder, together with potassium bromide, in a mortar and placed into a powder tablet machine to form tablets. Spectra were recorded on a Bruker Hyperion spectrometer by averaging signals over 32 scans in the wavenumber range of 4000−800 cm^−1^.

Wide angle X-ray diffractometer (WAXD) measurement. Profiles of the polyester samples were in-situ measured on a Rigaku 007HF diffractometer using a Mo *K*_α_ radiation source (λ = 0.7093 Å). The samples were raised to the target temperature at a rate of 10 °C/min and held for 3 min before testing. Data were collected in the 2θ interval from 5° to 25° with a scanning rate of 2°/min and a scanning step of 0.01°.

Proton nuclear magnetic resonance (^1^H NMR) measurement. The ^1^H NMR spectra of samples were measured on JNM-ECA 600M with chloroform-*d* (CDCl_3_) as the solvent and tetramethylsilane (TMS) as the standard.

## 4. Conclusions

In this study, crystalline/crystalline polymer blends of PBS/PBA were fabricated through an approach of co-inclusion complexation with urea molecules and investigated using DSC, WAXD, NMR and FTIR. It is demonstrated that the PBA chains were co-crystallized into PBS extended-chain crystals in the coalesced blends and the co-crystallized content increases with the initial blending ratio of the PBA. The PBA chains playing as defects mainly induced lattice expansion along the *a*-axis and the melting point of the PBS extended-chain crystal gradually declined from 134.3 °C to 124.2 °C with increasing the PBA content. In addition, when the co-crystals were soaked in tetrahydrofuran, some of the PBA chains were extracted out, leading to the damage of the correlative PBS extended-chain crystals. This study shows that co-inclusion complexation with small molecules could be an effective way to promote co-crystallization in polymer blends.

## Figures and Tables

**Table 1:** The mole ratios of PBA/PBS in the coalesced blends before and after THF soaking treatment.

Feeding (before)	^1^H-NMR (after)
10/90	3.4/96.6
20/80	3.7/96.3
30/70	4.0/96.0
40/60	6.1/93.9

**Table 2:** PBS lamellar crystals proportion and Tm of co-crystals of coalesced PBS/PBA blends before and after being soaked in THF.

PBS/PBA	PBS Lamellar Crystal Proportion (%)		*T*_m_ of Co-Crystal (°C)	
	before	after	before	after
90/10	10.9	12.3	132.8	132.9
80/20	18.0	21.9	131.9	132.2
70/30	21.8	23.1	126.1	129.4
60/40	23.8	31.5	124.0	128.4

## Data Availability

Data is contained within the article or Supplementary Material.

## Supplementary Materials

The following supporting information can be downloaded at https://www.mdpi.com/article/10.3390/molecules28104091/s1, Figure S1: DSC heating curves of PBS/PBA/urea-IC at a rate of 10 °C/min. (i)–(v) indicate PBS, PBS/PBA-90/10, PBS/PBA-80/20, PBS/PBA-70/30 and PBS/PBA-60/40, respectively; Figure S2: DSC heating curves of simply-blended PBS/PBA (dash lines) and coalesced PBS/PBA (solid lines) blends after being cooled to −30 °C form 90 °C (A) and the corresponding enthalpy values and their ratios (B). (i)–(iii) indicate PBS/PBA-80/20, PBS/PBA-70/30 and PBS/PBA-60/40, respectively; Figure S3: Lorentz peak decoupling of DSC curves coalesced PBS/PBA blends before (A) and after (B) being soaked in THF. The enthalpy of PBS lamellar crystals is shadowed with blue. (i)–(iv) indicate the PBS/PBA-90/10, PBS/PBA-80/20, PBS/PBA-70/30 and PBS/PBA-60/40 samples, respectively.
