# Supplementary material for: Co-Crystallization between Aliphatic Polyesters through Co-Inclusion Complexation with Small Molecule
Source: Molecules. 2023 May 15;28(10):4091. doi: 10.3390/molecules28104091 (PMC10222332; doi:10.3390/molecules28104091)
Supplement: Supplementary file 1 [file molecules-28-04091-s001.zip › molecules-2353911-supplementary.pdf]

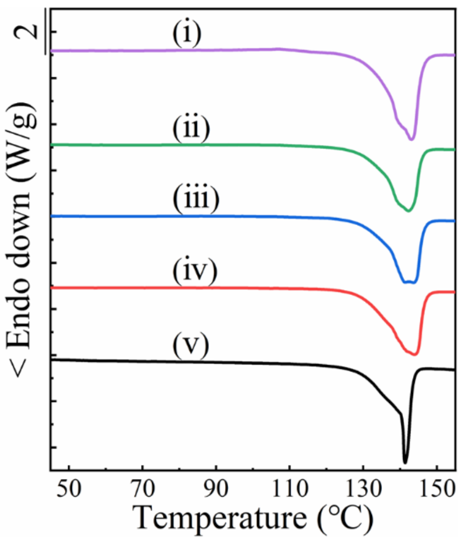

Figure S1. DSC heating curves of PBS/PBA/urea-IC at a rate of 10 °C/min. (i)–(v) indicate PBS, PBS/PBA-90/10, PBS/PBA-80/20, PBS/PBA-70/30, and PBS/PBA-60/40, respectively.

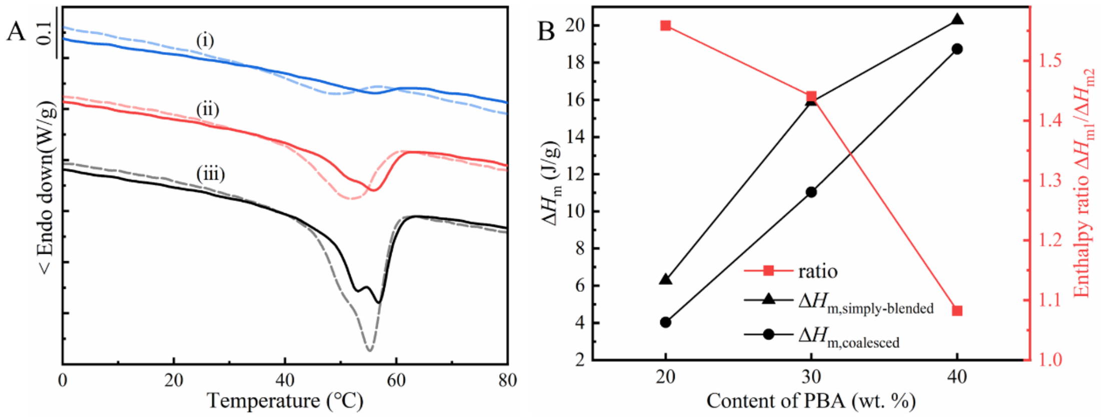

Figure S2. DSC heating curves of simply-blended PBS/PBA (dash lines) and coalesced PBS/PBA (solid lines) blends after being cooled to –30 °C form 90 °C (A), and the corresponding enthalpy values and their ratios (B). (i)–(iii) indicate PBS/PBA-80/20, PBS/PBA-70/30, and PBS/PBA-60/40, respectively.

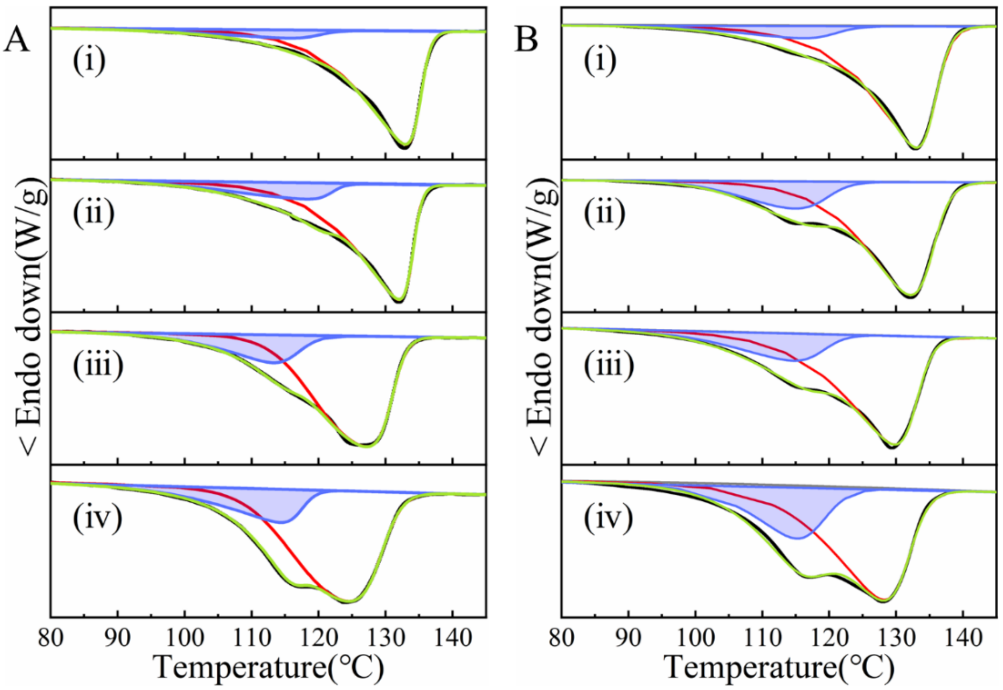

Figure S3. Lorentz peak decoupling of DSC curves coalesced PBS/PBA blends before (A) and after (B) being soaked in THF. The enthalpy of PBS lamellar crystals is shadowed with blue. (i)–(iv) indicate the PBS/PBA-90/10, PBS/PBA-80/20, PBS/PBA-70/30, and PBS/PBA-60/40 samples, respectively.
